# An Overview of Factors Influencing Psychiatric Out-Patient Satisfaction at a Tertiary Care Hospital in Pakistan

**DOI:** 10.7759/cureus.25834

**Published:** 2022-06-10

**Authors:** Fatima Tahir, Muneeb Ahmad, Kiran Ishfaq

**Affiliations:** 1 Department of Psychiatry, Jinnah Hospital, Lahore, PAK

**Keywords:** psychiatric outpatient satisfaction, cultural beliefs, patient satisfaction, socio-demographics, mental healthcare, psychiatry

## Abstract

Objectives

Patient satisfaction is now becoming the assessment criterion for the quality of health care services provided to patients with mental health issues; therefore, this study aimed to quantify patient satisfaction in the psychiatric outpatient department of Jinnah Hospital, Lahore, Pakistan, and assess the effects of socio-demographic factors and cultural and ethical beliefs on patient satisfaction.

Methods

This is a cross-sectional, observational study with a sample size of 386 patients, using a simple random sampling technique. Patients older than 14 years were included in this study. A questionnaire comprising demographics and cultural and ethical beliefs using the Cultural Attitudes toward Healthcare and Mental Illness Questionnaire, and satisfaction rates using the Psychiatric Out-Patient Experience Questionnaire (POPEQ), was designed for the research project.

Results

The mean age ± SD was 31.2 ± 12.2 years. The POPEQ demonstrated a mean satisfaction score of 3.11 ± 0.90. The majority of the population considered stress (54.4%), family issues (33.4%), and medical illness (33.4%) as the cause of their mental illness. In comparison, the preferable type of treatment for most patients was medication (75.1%) and counseling (36.0%). Among socio-demographic characteristics, education was inversely related to satisfaction (p<0.01). The patients who believed medications to be their preferred treatment for their mental illness were most satisfied (p < 0.01).

Conclusion

This study demonstrates high overall satisfaction rates with psychiatric outpatient services. However, no significant association between sociodemographic characteristics and satisfaction levels was established except for the education status of the patients and their preferred method of treatment. The study did not reveal any influence of cultural beliefs on the degree of satisfaction of patients.

## Introduction

The global burden of mental health disorders has increased exponentially in the last few decades, with a significant increase in disability-affected life years (DALY) [1]. Despite the escalating incidence of mental health disorders in low- and middle-income countries like Pakistan, mental health services remain insufficient. There is one psychiatrist for a population of 0.5-1 million, with a similar ratio for psychologists. The five major mental health hospitals in big cities of Pakistan provide 1.9 beds: 100,000 population [2]. Another alarming fact is that community-based outreach services are practically non-existent. Therefore, the quality of care provided by the existing healthcare services needs careful evaluation, so extended plans can be formulated for the future.

In recent years, patient satisfaction with psychiatric services has become the cornerstone of quality assessment as it is important to take into account the opinion of its users to improve the standard of health care. Many developed countries emphasize patient-centered care as the primary goal of their services [3]. There has also been a surge in research regarding patient satisfaction, however, there is minimal data available from developing countries like Pakistan. Thimm et al. reported that active involvement of psychiatric patients in their management such as decision making, setting treatment goals, and termination of treatment was strongly associated with the satisfaction of patients [4]. Patient expectations from a mental health service define their satisfaction and consequently, the nature of their experience. Therefore, mental health care providers are expected to deliver customized care to each individual to meet those expectations.

There is a multitude of socio-demographic and clinical associations thought to influence patient satisfaction, but the results differ among studies. Being a female, being of older age, being married, and being employed were associated with higher levels of satisfaction [5]. Self-perception of being physically and mentally sound was also associated with increased satisfaction [6]. In a study in Malawi about the impact of living areas on satisfaction, the rural population was found to be significantly less satisfied than the urban population [7]. In contrast, several other studies found no significant association between socio-demographic or clinical factors and patient satisfaction [8]. Furthermore, satisfaction rates may differ among patients receiving treatment from the same mental healthcare service. Some deficiencies on part of the mental health care team have also been reported, which need to be addressed, including inadequate treatments and limited use of guidelines [9-14]. Additionally, there is also some disparity between services in different geographical regions of the world [15].

Patients suffering from mental ailments are stigmatized and discriminated against worldwide, contributing further to their poor quality of life [10,16-18]. For most patients and their families, presentation to mental health service is their last hope to improve their quality of living and overcome the psychological barriers maligning their daily lives. In an outpatient department, patient satisfaction with their mental health care experience plays a critical role in their compliance to treatment, regular follow-up, and outcome. Thus, quality assessment is needed to identify the gaps in the mental health care system and identify the key elements leading to patient dissatisfaction.

Cultural and ethical beliefs in a region determine the attitude of patients towards mental health services [15]. The health belief model of an individual shapes his interaction with the physician and compliance with treatment. Hence, it indirectly influences the effectiveness of a health care system to treat its patients. The primary objective of this study was to quantify the overall patient satisfaction and assess the influence of demographic, cultural, and ethical beliefs on the satisfaction levels of patients presenting in the psychiatric outpatient department of Jinnah Hospital, Lahore, Pakistan.

## Materials and methods

Study design and procedure

This observational, cross-sectional study was designed to measure the patient satisfaction with psychiatric out-patient services of Jinnah Hospital, a tertiary care hospital located in the center of Lahore, Pakistan, over two months. Investigators who were working as interns in the same department handed out questionnaires to patients after their consultations. Treating psychiatrists were not aware of the feedback given by their patients, eliminating any chance of interference in the treatment process of the patients. Informed consent was taken in written form after explaining the purpose of this research. Patients were guaranteed that being a part of this research will not affect their treatment. Approval for this study was taken from the Ethical Review Board of Allama Iqbal Medical College/Jinnah Hospital, Lahore with approval number: 22/14/01/2021/S2 ERB. The data collection period lasted from February 14, 2021, to April 10, 2021.

Instruments

The questionnaire was broken down into four sections: section one comprised sociodemographic characteristics including sex, age, employment status, monthly income, marital status, number of children, area of residence, and number of family members at home; section two assessed cultural beliefs regarding psychiatric services using modified a Cultural Attitudes toward Healthcare and Mental Illness Questionnaire in the form of multiple-choice questions; section three measured patient satisfaction through Psychiatric Out-Patient Experiences Questionnaire (POPEQ), the 11 items of which were ranked on a five-point Likert scale from ‘not at all' (0) to ‘to a very high degree’ (4) giving a single-index answer; and section four included information about the treatment plan of the patient, whether drugs or therapy. The Cultural Attitudes toward Healthcare and Mental Illness Questionnaire, developed for the Primary Care Research in Substance Abuse and Mental Health for the Elderly (PRISM-E) [19], was modified to be used for this research. This questionnaire was used to demonstrate the beliefs of patients regarding the cause of their mental illness, their preferences for the type of treatment, their autonomy regarding treatment decisions, and the desirable attributes of their treating psychiatrist/psychologist. POPEQ is a validated and reliable instrument with good test-retest reliability [20]. It can be subdivided into three subscales: quality of clinical interaction (six items), the outcome of the treatment (three items), and information provision (two items). Internal consistency of POPEQ was assessed by Olsen et al. (2010), which was found to be high with Cronbach's alpha and test-retest reliability above 0.9 and variance around 50% [21]. This patient satisfaction scale has been reported to have moderate-to-excellent psychometric properties. The questionnaire, originally in English, was translated into the local language (Urdu) and back-translated into English taking assistance from the expertise of a local translator. The questionnaire was read out to those individuals who had no formal education at school and were unable to read and write. Their answers were recorded by the investigators on the questionnaire form. 

Study sample

The sample size was calculated by using a single proportion formula for continuous data i-e; normally-distributed, where σ is for the unknown variance or SD (0.5), Z is the reliability coefficient at 95%CI (1.96), d is the size of difference to detect the minimal effect of interest. With the margin of error of 0.05 and 5% incomplete filling of the questionnaire, our sample size was calculated to be 410-24=386. Simple random sampling technique was used. Eligible individuals were all adolescents (≥14 years) and older adults with no cognitive or physical impairment. All children less than 14 years old and all individuals who were physically or mentally incompetent were excluded. 

Data analysis

Data were analyzed using IBM SPSS Statistics for Windows, Version 23.0 (Released 2015, IBM Corp., Armonk, New York, United States). Two-tailed Pearson’s chi-square tests were used with significance levels set at 0.05 and 0.01 for comparing frequencies.

## Results

Socio-demographic characteristics of the patients

The socio-demographic characteristics of 386 individuals (42.2% males, 57.8% females) participating in this study for one month are given in Table 1. Mean age ± SD was 31.2 ± 12.2 years and ranged from 14 to 70 years. The majority (80.3%) of the participants were living in urban areas, 6.5% in suburban areas, and 13.2% in rural areas. Of the participants, 11.7% were illiterate (unable to read or write), while 24.8% were graduates with bachelor's, master's, and higher degrees. The rest of the population had completed their primary or secondary education. The marital status of the candidates showed that 41.2% were unmarried, 51.2% were married, and 7.5% were divorced or widowed. Regarding the households, 45.3% of the participants were living in a joint family, while 54.7% were living as a nuclear family with an average of 8.57 individuals per household.

**Table 1:** Demographics

Variable	Mean (SD)	Frequency (%)
Age	31.2 (12.2)	
Sex		
i. Male		163 (42.2)
ii. Female		223 (57.8)
Living Area		
i. Urban		310 (80.3)
ii. Sub-urban		25 (6.5)
iii. Rural		51 (13.2)
Education		
i. Illiterate		45 (11.7)
ii. Islamic Education		16 (4.1)
iii. Primary		19 (4.9)
iv. Middle		75 (19.4)
v. Secondary		85 (22.0)
vi. Higher Secondary		50 (13.0)
vii. Graduate (Bachelors/Masters/PhD)		96 (24.8)
Employment Status		
i. Employed		119 (30.8)
ii. Unemployed		70 (18.1)
iii. Student		78 (20.2)
iv. Housewife		119 (30.8)
Marital Status		
i. Single		159 (41.2)
ii. Married		198 (51.3)
iii. Widowed		11 (2.8)
iv. Divorced		18 (4.7)
Family Type		
i. Nuclear		211 (54.7)
ii. Joint		175 (45.3)
Siblings	5.3 (2.2)	
Children	3.1 (2.0)	
Total individuals in the house	8.5 (4.8)	

Patient satisfaction

The POPEQ demonstrated a mean satisfaction score of 3.11 ± 0.90 (Table 2). Further categorization in three subscales revealed average satisfaction scores to be 3.16, 3.23, and 2.70 for change in mental illness since the start of treatment, interaction with the doctor, and information regarding illness and treatment, respectively. 

**Table 2:** Psychiatric Out-Patient Experience Questionnaire (POPEQ) scores

	Mean (SD)
Change	3.16 (0.88)
Interaction	3.23 (0.90)
Information	2.70 (1.39)
Total	3.11 (0.90)

Among socio-demographic characteristics, education was inversely related to the satisfaction level of the patients (p<0.01), indicating that the more educated the patient, the less satisfied he/she is likely to be by the services provided at the psychiatry out-patient department (Table 3). Increasing age and the number of children also had a slight direct impact on the degree of satisfaction. 

**Table 3:** Relationship between satisfaction score and demographics ** Correlation is significant at the 0.01 level (2-tailed); * Correlation is significant at the 0.05 level (2-tailed); c Cannot be computed because at least one of the variables is constant

Variables	Correlation	Change	Interaction	Information	Total
Sex	Pearson Correlation	0.063	0.016	0.020	0.032
	Sig. (2-tailed)	0.217	0.753	0.693	0.526
Age	Pearson Correlation	0.117^*^	0.106^*^	0.087	0.115^*^
	Sig. (2-tailed)	0.021	0.037	0.087	0.024
Living type	Pearson Correlation	-0.013	-0.015	-0.005	-0.013
	Sig. (2-tailed)	0.794	0.765	0.917	0.801
Education	Pearson Correlation	-0.139^**^	-0.196^**^	-0.108^*^	-0.175^**^
	Sig. (2-tailed)	0.006	0.000	0.034	0.001
Marital status	Pearson Correlation	0.096	0.063	0.053	0.076
	Sig. (2-tailed)	0.059	0.217	0.301	0.135
Children	Pearson Correlation	0.126	0.146^*^	0.100	0.143^*^
	Sig. (2-tailed)	0.059	0.029	0.137	0.033
Siblings	Pearson Correlation	0.040	0.019	0.048	0.036
	Sig. (2-tailed)	0.438	0.703	0.342	0.478
Family type	Pearson Correlation	-0.030	0.004	-0.068	-0.026
	Sig. (2-tailed)	0.559	0.937	0.184	0.605
Total individuals in the house	Pearson Correlation	0.030	0.003	0.011	0.013
	Sig. (2-tailed)	0.562	0.948	0.830	0.796

Cultural and ethical beliefs

The majority of the population considered stress (54.4%), family issues (33.4%), and medical illness (33.4%) as the cause of their mental illness (Table 4). The preferable type of treatment for most patients was medication (75.1%) including pills, injectables, or oral solutions, and counseling (36.0%). Most individuals preferred to talk about their mental health with their family members (55.4%), including parents and siblings, and their treating psychiatrist (37.8%). A significant portion of the population (42.5%) stated that they make mental health care decisions on their own. The patients who believed oral medications to be their preferred treatment for their mental illness were most satisfied (p < 0.01). In contrast, the patients preferring psychological counseling were left significantly unsatisfied (p < 0.01).

**Table 4:** Cultural influences on mental health ** Correlation is significant at the 0.01 level (2-tailed); * Correlation is significant at the 0.05 level (2-tailed); c Cannot be computed because at least one of the variables is constant POPEQ: Psychiatric Out-Patient Experience Questionnaire

Variables (questionaire)	Frequency (%)	Correlation with POPEQ	Change	Interaction	Information	Total
What, in your opinion, is the cause of your mental illness?						
Stress/worry	210 (54.4%)	Pearson Correlation	0.087	0.070	0.082	0.085
		Sig. (2-tailed)	0.087	0.170	0.108	0.095
Loss (family, friends)	69 (17.9%)	Pearson Correlation	0.010	0.005	0.021	0.011
		Sig. (2-tailed)	0.843	0.929	0.678	0.823
Lack of pleasurable activities	67 (17.4%)	Pearson Correlation	-0.114^*^	-0.098	-0.050	-0.099
		Sig. (2-tailed)	0.025	0.054	0.327	0.052
Family issues	129 (33.4%)	Pearson Correlation	-0.007	0.018	0.016	0.012
		Sig. (2-tailed)	0.887	0.731	0.752	0.818
Political stress	10 (2.6%)	Pearson Correlation	-.0074	-0.088	-0.030	-0.076
		Sig. (2-tailed)	0.144	0.084	0.560	0.134
Safety issues	29 (7.5%)	Pearson Correlation	-0.025	-0.046	0.004	-0.030
		Sig. (2-tailed)	0.624	0.370	0.938	0.555
Medical illness	129 (33.4%)	Pearson Correlation	-0.068	-0.021	0.057	-0.012
		Sig. (2-tailed)	0.186	0.686	0.260	0.807
Infectious disease	1 (0.3%)	Pearson Correlation	0.029	0.033	0.047	0.040
		Sig. (2-tailed)	0.573	0.512	0.354	0.438
Nutritional deficiency	32 (8.3%)	Pearson Correlation	-0.047	-0.049	-0.007	-0.041
		Sig. (2-tailed)	0.356	0.333	0.890	0.426
Genetic	24 (6.2%)	Pearson Correlation	-0.062	-0.064	-0.107^*^	-0.083
		Sig. (2-tailed)	0.227	0.208	0.036	0.105
Chemical imbalance	53 (13.7%)	Pearson Correlation	-0.014	-0.029	-0.026	-0.027
		Sig. (2-tailed)	0.784	0.575	0.609	0.600
Spirit/Psyche	68 (17.6%)	Pearson Correlation	0.004	-0.041	-0.009	-0.023
		Sig. (2-tailed)	0.941	0.420	0.855	0.651
Disturbance of body, mind, and spirit	61 (15.8%)	Pearson Correlation	-0.040	-0.054	0.013	-0.035
		Sig. (2-tailed)	0.431	0.288	0.802	0.492
Something wrong you did in the past	39 (10.9%)	Pearson Correlation	-0.087	-0.058	-0.142^**^	-0.097
		Sig. (2-tailed)	0.087	0.256	0.005	0.057
Supernatural (witchcraft, hexes)	33 (8.5%)	Pearson Correlation	0.036	0.006	-0.012	0.012
		Sig. (2-tailed)	0.482	0.900	0.820	0.814
Environment/culture	43 (11.1%)	Pearson Correlation	-0.062	-0.055	0.040	-0.035
		Sig. (2-tailed)	0.226	0.281	0.439	0.491
Moving to a different place	26 (6.7%)	Pearson Correlation	-0.059	-0.006	0.005	-0.020
		Sig. (2-tailed)	0.244	0.905	0.922	0.689
Cultural differences	17 (4.4%)	Pearson Correlation	-0.013	0.027	0.018	0.016
		Sig. (2-tailed)	0.806	0.602	0.720	0.761
Adjusting to a different culture	30 (7.8%)	Pearson Correlation	-0.015	0.001	0.023	0.003
		Sig. (2-tailed)	0.762	0.981	0.648	0.959
Drugs	4 (1.0%)	Pearson Correlation	0.029	0.014	0.040	0.028
		Sig. (2-tailed)	0.574	0.777	0.434	0.585
None of these	19 (4.9%)	Pearson Correlation	0.011	0.033	-0.098	-0.009
		Sig. (2-tailed)	0.835	0.523	0.055	0.863
Treatment preferences						
Pills/medications	290 (75.1%)	Pearson Correlation	0.222^**^	0.264^**^	0.190^**^	0.258^**^
		Sig. (2-tailed)	0.000	0.000	0.000	0.000
Herbal remedies	19 (4.9%)	Pearson Correlation	-0.089	-0.074	-0.025	-0.071
		Sig. (2-tailed)	0.081	0.149	0.627	0.165
Counselling	139 (36.0%)	Pearson Correlation	-0.220^**^	-0.243^**^	-0.131^**^	-0.228^**^
		Sig. (2-tailed)	0.000	0.000	0.010	0.000
Group counselling	19 (4.9%)	Pearson Correlation	-0.094	-0.091	-0.081	-0.098
		Sig. (2-tailed)	0.066	0.074	0.114	0.055
Alternative therapies	29 (7.5%)	Pearson Correlation	-0.099	-0.071	-0.035	-0.076
		Sig. (2-tailed)	0.051	0.166	0.496	0.135
Spiritual advice	50 (13.0%)	Pearson Correlation	0.083	0.051	0.032	0.061
		Sig. (2-tailed)	0.101	0.316	0.532	0.229
Who would you talk to about your mental health/substance abuse issues?						
Spouse	74 (19.2%)	Pearson Correlation	0.113^*^	0.062	0.077	0.088
		Sig. (2-tailed)	0.026	0.225	0.131	0.085
Family member living with you0	214 (55.4%)	Pearson Correlation	0.022	0.053	0.018	0.039
		Sig. (2-tailed)	0.662	0.298	0.731	0.445
Family member not living with you	25 (6.5%)	Pearson Correlation	-0.031	-0.014	0.041	-0.005
		Sig. (2-tailed)	0.548	0.780	0.426	0.920
Friend	101 (26.2%)	Pearson Correlation	0.017	0.068	0.056	0.057
		Sig. (2-tailed)	0.736	0.180	0.270	0.265
Healer	38 (9.8%)	Pearson Correlation	-0.037	-0.038	-0.048	-0.044
		Sig. (2-tailed)	0.467	0.452	0.343	0.391
Psychiatrist	146 (37.8%)	Pearson Correlation	-0.012	0.027	0.008	0.012
		Sig. (2-tailed)	0.808	0.602	0.874	0.816
Medical doctor	29 (7.5%)	Pearson Correlation	-0.058	-0.035	0.025	-0.027
		Sig. (2-tailed)	0.252	0.494	0.623	0.597
Social worker	11 (2.8%)	Pearson Correlation	-0.151^**^	-0.153^**^	-0.153^**^	-0.168^**^
		Sig. (2-tailed)	0.003	0.003	0.003	0.001
12-step programs	0 (0.0%	Pearson Correlation	.^c^	.^c^	.^c^	.^c^
		Sig. (2-tailed)	.	.	.	.
Someone from mosque	33 (8.5%)	Pearson Correlation	-0.048	-0.004	0.008	-0.014
		Sig. (2-tailed)	0.345	0.935	0.871	0.791
Religious spiritual leader	31 (9.0%)	Pearson Correlation	-0.046	-0.054	-0.108^*^	-0.072
		Sig. (2-tailed)	0.368	0.294	0.034	0.156
Alternative care provider	9 (2.3%)	Pearson Correlation	-0.108^*^	-0.094	-0.023	-0.086
		Sig. (2-tailed)	0.035	0.064	0.657	0.091
None	34 (8.8%)	Pearson Correlation	-0.056	-0.055	-0.049	-0.058
		Sig. (2-tailed)	0.270	0.285	0.339	0.252
Who makes your mental health care decisions?						
You	164 (42.5%)	Pearson Correlation	-0.064	-0.104^*^	-0.070	-0.093
		Sig. (2-tailed)	0.210	0.041	0.172	0.068
Spouse	97 (25.1%)	Pearson Correlation	0.092	0.100^*^	0.046	0.092
		Sig. (2-tailed)	0.071	0.049	0.372	0.07
Doctor	112 (29.0%)	Pearson Correlation	-0.019	-0.014	-0.034	-0.022
		Sig. (2-tailed)	0.708	0.785	0.500	0.664
Family member other than spouse	89 (23.1%)	Pearson Correlation	0.018	0.058	0.052	0.050
		Sig. (2-tailed)	0.722	0.258	0.309	0.326
Someone else	21 (5.4%)	Pearson Correlation	-0.068	0.008	0.018	-0.009
		Sig. (2-tailed)	0.185	0.877	0.724	0.867

The most desirable characteristics of the healthcare provider were: speaking the same language as the patient, understanding the culture of the patient (Table 5), and being open to different treatment options; however, none of these factors were found to be significant (p>0.05).

**Table 5:** Preferred characteristics of health care provider ** Correlation is significant at the 0.01 level (2-tailed); * Correlation is significant at the 0.05 level (2-tailed); c Cannot be computed because at least one of the variables is constant POPEQ: Psychiatric Out-Patient Experience Questionnaire

		Frequency (%)	Correlation with POPEQ	Change	Interaction	Information	Total
Speaking same language	Disagree	221 (57.3%)	Pearson Correlation	0.051	0.047	0.061	0.057
	Neither Agree nor Disagree	140 (36.3%)					
	Agree	25 (6.5%)	Sig. (2-tailed)	0.315	0.352	0.229	0.260
Being same racial/ethnic group	Disagree	386 (100%)	Pearson Correlation	0.065	0.082	0.094	0.089
	Neither Agree nor Disagree	0 (0.0%)					
	Agree	0 (0.0%)	Sig. (2-tailed)	0.201	0.108	0.065	0.082
Being same gender	Disagree	295 (76.4%)	Pearson Correlation	0.034	0.036	0.015	0.032
	Neither Agree nor Disagree	86 (22.3%)					
	Agree	5 (1.3%)	Sig. (2-tailed)	0.504	0.479	0.774	0.535
Being same age	Disagree	386 (100%)	Pearson Correlation	0.003	0.018	-0.013	0.006
	Neither Agree nor Disagree	0 (0.0%)					
	Agree	0 (0.0%)	Sig. (2-tailed)	0.958	0.730	0.7960	0.901
Being open to different treatment	Disagree	304 (78.7%)	Pearson Correlation	0.028	0.016	0.042	0.030
	Neither Agree nor Disagree	72 (18.7%)					
	Agree	10 (2.5%)	Sig. (2-tailed)	0.580	0.753	0.413	0.562
Understanding your culture	Disagree	174 (45.0%)	Pearson Correlation	-0.051	0.004	0.008	-0.011
	Neither Agree nor Disagree	166 (43.0%)					
	Agree	46 (11.9%)	Sig. (2-tailed)	0.316	0.943	0.882	0.835

## Discussion

This study assessed the degree of satisfaction of psychiatric outpatients by qualitative measurement tools, revealing that majority of the patients were highly satisfied with the services provided by the psychiatric department. As compared to previous studies [4,8], no statistically significant association between patient satisfaction and socio-demographic characteristics (age, gender, marital status, occupation, employment status, area of residence, and housing situation) was established except for the education status of patients, which showed that higher education level was linked to greater dissatisfaction. One possible reason is that higher education leads to higher expectations from mental health care providers and significantly impacts patient satisfaction. These results are consistent with another study carried out in Nigeria by Obayi et al., in which education was found to be inversely related to patient satisfaction [5].

Assessment of patient satisfaction by POPEQ showed that patients were most satisfied with their ‘interaction’ with the psychiatrist and psychologist, followed by the ‘change’ in their illness since the start of treatment, indicating that quality doctor-patient interaction and improvement in symptoms had a positive impact on patient satisfaction. This study also revealed that a significant percentage of patients were dissatisfied with the information given to them about their disease and its treatment options. Many other studies have shown that information about the disease, treatment options, and psychoeducation has a profound effect on patient satisfaction [3]. Lack of involvement in mental health care decisions leads to a loss of mutual trust between a patient and the treating physician and declines the quality of interaction [4].

The respondents were asked about their opinion regarding the triggering and contributing factors to their mental illness. Most patients considered stress, family issues, and physical illnesses as an important cause of their mental illness rather than superstitious beliefs like witchcraft and spirits and, thus, relied more on psychotherapy and medications for treatment. These results are in contrast to a study conducted in India by Kate et al. (2012), which revealed that two-thirds of the population believed sorcery and evil spirits were a probable cause of mental illnesses [22]. It may be due to a bias as patients seeking help from a hospital may not believe in evil spirits and sorcery causing mental health problems. Our findings establish that the patients who believe to be cured by medications are most satisfied with their healthcare provider and those who expect to be cured by counseling are least satisfied. However, the authors believe that due to the potential for dependence and significant adverse effects of psychiatric medications, patients should be educated more about the benefits of non-pharmacological methods by their treating doctor. Several pieces of research have shown comparable efficacy of cognitive therapy to medications for treating depression [23], making it an acceptable alternative. Family and friends often play a supportive role in assisting patients in seeking help regarding mental illness, but it was interesting to note that the majority of the study population decided to seek help on their own. The preferable attributes of health care providers according to the patients were speaking the same language, understanding the culture of patients, and keeping multiple treatment options in view. These findings are similar to a recent study by Taylor (2020), which revealed that clients prefer providers that are similar to themselves in various aspects [24].

The strengths of this study include a large sample size. Selection bias was minimized by using simple random sampling and was representative of the population presenting in the outpatient psychiatry department. Another strength was the utilization of validated and standardized questionnaires. To reduce observer bias, the collection of data was done by interns who were not directly involved in patient treatment, and patients were encouraged to express their views freely.

Limitations of this study include a higher tendency towards reporting a positive response due to the ongoing nature of treatment and false interpretation of the questions as they were translated from English.

## Conclusions

This study demonstrates high overall satisfaction rates with psychiatric outpatient services, which indicates satisfactory services being provided at tertiary care hospitals. However, no significant association between socio-demographic characteristics and satisfaction levels was established, except for the education status of the patients as higher education status increased the likelihood of dissatisfaction. Most patients considered stress to be the cause of their mental illness and expected to be treated with medications.

Future studies should focus on determining patient satisfaction scores at primary and secondary healthcare services, and finding other parameters that impact the satisfaction of psychiatric patients. Moreover, the possible methods to be adopted by the doctors to improve patient satisfaction should be highlighted.
